# Primary intraosseous squamous cell carcinoma of the mandible: a case with atypical imaging features

**DOI:** 10.1259/bjrcr.20150276

**Published:** 2016-11-02

**Authors:** João Lopes Dias, Alexandra Borges, Rafaela Lima Rego

**Affiliations:** ^1^Department of Radiology, Hospital de São José, Centro Hospitalar de Lisboa Central, Lisbon, Portugal; ^2^Department of Radiology, Instituto Português de Oncologia de Lisboa Francisco Gentil, Lisbon, Portugal; ^3^Department of Pathology, Instituto Português de Oncologia de Lisboa Francisco Gentil, Lisbon, Portugal

## Abstract

Primary intraosseous squamous cell carcinoma is a rare malignant tumour that exclusively arises within the jaws. Its diagnosis requires an appropriate clinical, imaging and histological correlation. The exclusion of primary oral mucosa lesions and metastatic disease is mandatory. We report an atypical imaging appearance of this uncommon entity, characterized by new bone formation and periosteal reaction that resemble sarcomatous or malignant odontogenic tumours. A comprehensive discussion on the embryological principles of primary intraosseous squamous cell carcinoma is also provided.

## Background

Primary intraosseous squamous cell carcinoma (PIOSCC) is a rare malignant tumour that arises within the jaws and derives from odontogenic epithelial remnants.^1–4^ Owing to its rarity, clinical and radiological diagnostic criteria are not well established. Varied nomenclature has been used for this tumour since Loos first described it in 1913.^2^ Terminologies such as intra-alveolar epidermoid carcinoma or primary intra-alveolar epidermoid carcinoma have fallen in disuse and the most recent World Health Organization Classification of Tumours in 2005 used the term PIOSCC for this tumour.^2^ According to this classification, three subcategories of PIOSCC may be found: (1) solid tumours that invade the marrow spaces and induce osseous resorption; (2) squamous cancer arising from the lining of an odontogenic cyst; (3) squamous cell carcinoma (SCC) in association with other benign epithelial odontogenic tumours.^4^

Clinical and imaging features are non-specific and the first impression of both clinicians and radiologists usually favours the diagnosis of alveolar or gingival SCC with bone invasion, or metastatic SCC.^5–7^ We report a PIOSCC with atypical imaging findings on both CT and MRI. A discussion on the relevant embryological and histological features is also provided.

## Case report

A 42-year-old male presented to the Department of Head and Neck Surgery of a tertiary oncological centre because of right mandibular swelling and trismus. The patient had already been admitted to a secondary care hospital 3 months earlier with complaints of right mandibular discomfort and slight tumefaction. A biopsy was then performed and the diagnosis of ameloblastic carcinoma was made histologically. The patient was otherwise healthy with no significant past medical history, including alcohol, smoking or tobacco abuse.

A complete head and neck examination revealed a painless, firm and fixed right mandibular mass with no cutaneous inflammatory signs. No ulcers or mucosal lesions were found in the oral cavity. Laboratory evaluation, chest radiograph and respiratory function tests were unremarkable. The patient underwent bronchofibroscopy, which revealed only mild laryngeal hyperaemia.

**Figure 1.**
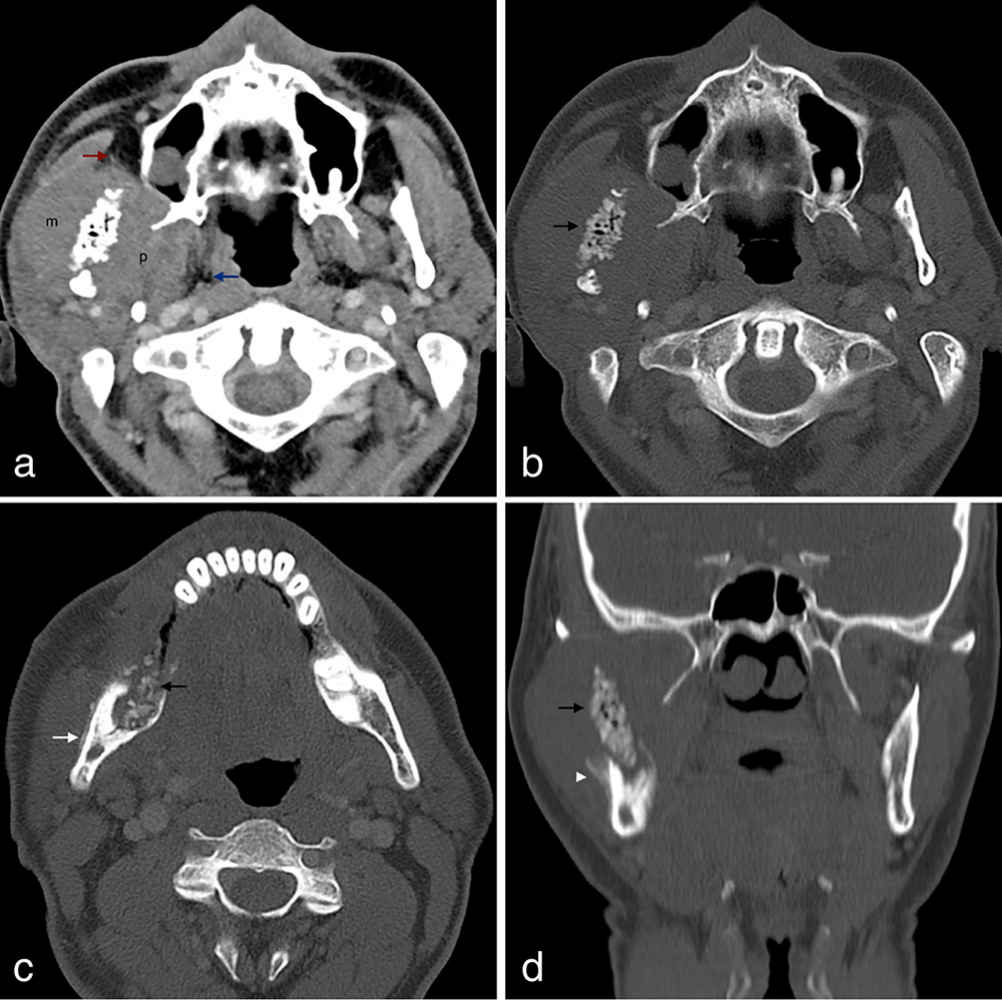
(a) Axial enhanced CT image in soft tissue window settings reveals a large right mandibular tumour consisting of a soft tissue component surrounding a markedly irregular and sclerotic mandibular ramus with some gas bubbles within the medullary cavity. The soft tissue component invades the masseter muscle (**m**) laterally and the pterygoid muscles (**p**) medially. The ipsilateral parapharyngeal space (blue arrow) and the Bichat’s fat pad (red arrow) are medially and anteriorly displaced, respectively. (b–d) Axial and coronal CT images in bone window settings better depict the heterogeneous bone involvement with a more irregular and sclerotic pattern in the mandibular ramus occupying the central portion of the tumour (black arrows) and a more expansive, remodelling pattern in the angle and posterior body with a ground-glass pattern of bone-forming matrix. Different patterns of periosteal reaction are depicted lengthwise in the mandibular ramus, including a thick regular deposition resembling an “onion skin” (white arrow) and a ruptured Codman triangle (white arrowhead).

**Figure 2.**
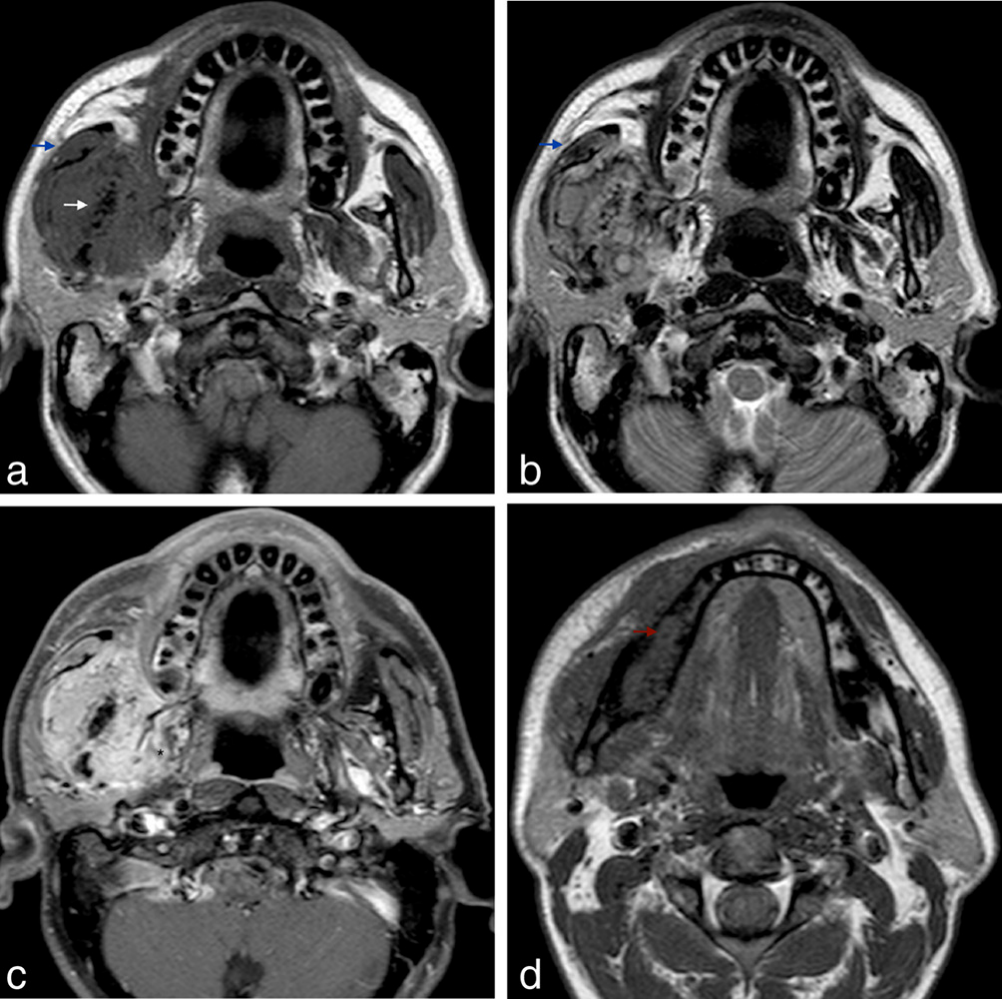
(a, b) Synchronized axial *T*_1_ weighted and *T*_2_ weighted MR images show multiple coalescent hypointense foci in the region of the mandibular ramus (white arrow) and a surrounding soft tissue component isointense and hyperintense in relation to the remaining masseter (blue arrows) on *T*_1_ weighted image and *T*_2_ weighted image, respectively. (**c**) Axial enhanced fat-suppressed *T*_1_ weighted MR image demonstrates avid tumour enhancement and better depicts the involvement of the pterygoid space (asterisk). (**d**) Axial *T*_1_ weighted MR image at a lower level shows the replacement of the normal fatty marrow by an expansive soft tissue mass expanding and remodelling the cortical bone (red arrow).

Both neck CT (Figure 1) and MRI (Figure 2) were performed, showing a large, solid tumour arising from the ramus and posterior body of the right mandible. The lesion extended to the surrounding soft tissues, with invasion of the masseter and medial pterygoid muscles and caused bulging of the buccal mucosa. The soft tissue component was hypointense on *T*_1_ weighted and hyperintense on *T*_2_ weighted MR images and showed avid enhancement after gadolinium administration on MR examination. CT scan disclosed striking sclerosis and irregularity of the mandibular ramus with some gas bubbles inside the medullary cavity and an expansive lytic component in the posterior body and angle with some bone-forming matrix inside. Prominent periosteal reaction was also identified, particularly in the outer cortical surface of the mandibular ramus with the typical pattern of a ruptured Codman triangle. No associated cystic lesion was found in the mandible. No enlarged lymph nodes were detected and the evaluation of the remaining cervical spaces was unremarkable. A thoracic CT scan was also performed, with no parenchymal lesions.

**Figure 3.**
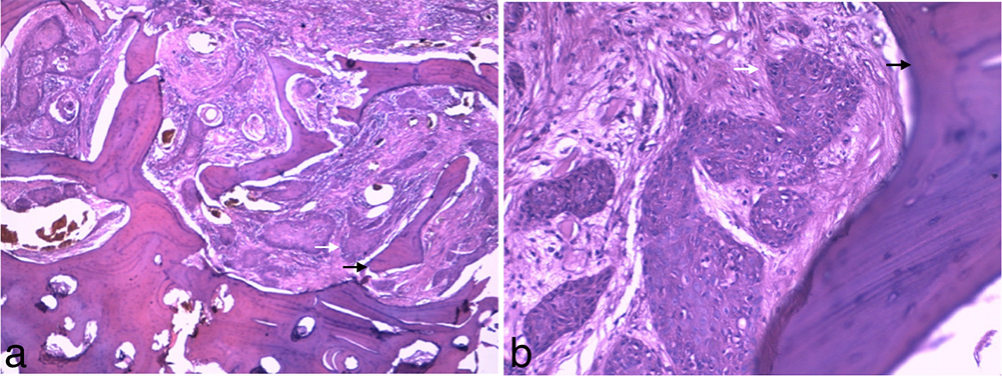
(a, b) Specimen from left hemimandibulectomy (haematoxylin and eosin stain, ×4 and ×20 magnification, respectively). Nests of neoplastic squamous cells (white arrow) may be seen infiltrating the trabecular bone (black arrow).

The patient underwent a right hemimandibulectomy and ipsilateral cervical lymph node dissection. Surgical resection also included the right submandibular gland and a segment of buccal mucosa that was swollen by the mandibular mass. Reconstruction was performed with free fibula graft. The surgical specimen included a large, white and solid tumour with 11 × 7.5 × 6.5 cm, corresponding to an invasive, moderately differentiated (G2) SCC (Figure 3). Focal positive margins were found at the medial surface of the specimen. The resected buccal mucosa, submandibular gland and lymph nodes had no neoplastic tissue. Taking into account the imaging staging examinations and the post-surgical histological report, the final TNM stage was stated as pT3 N0 Mx. After surgery, the patient underwent adjuvant chemotherapy (cisplatin-based regimen) and radiotherapy.

## Discussion

PIOSCC is a rare tumour with only a few cases reported in the literature. It is exclusively found within the jaws, the only bones that may give origin to both connective and epithelial tumours. The mandible is much more often involved than the maxilla.^8^ The association of the PIOSCC with odontogenic cysts has already been reported, particularly with residual and radicular cysts, and less frequently with dentigerous cysts, odontogenic keratocysts and lateral periodontal cysts.^7,9,10^ In rare cases, PIOSCC results from dedifferentiation of a benign ameloblastoma.^4^

Risk factors and causes are not well established, but since PIOSCC develops without initial communication with the oral mucosa, exogenous carcinogens such as tobacco and alcohol are unlikely to be involved.^7^ Other factors such as long-standing chronic inflammation and keratinization have been postulated to increase the risk of malignant transformation within an odontogenic cyst.^8^

A predilection for males (2:1) is described, as well as an increased incidence in the fifth decade.^11^ Symptoms are non-specific and depend on the location, size and aggressiveness of the tumour. Pain, swelling, sensory disturbance (due to inferior alveolar nerve involvement) and odontogenic disorders are commonly found.^11^

Bodner et al^9^analysed the clinical and pathological features of 116 PIOSCCs arising in odontogenic cysts, and reported a large majority of well-to-moderately differentiated tumours (85%). In the same study, the overall survival rate was 62% at 2 years and 38% at 5 years.^12^

**Figure 4.**
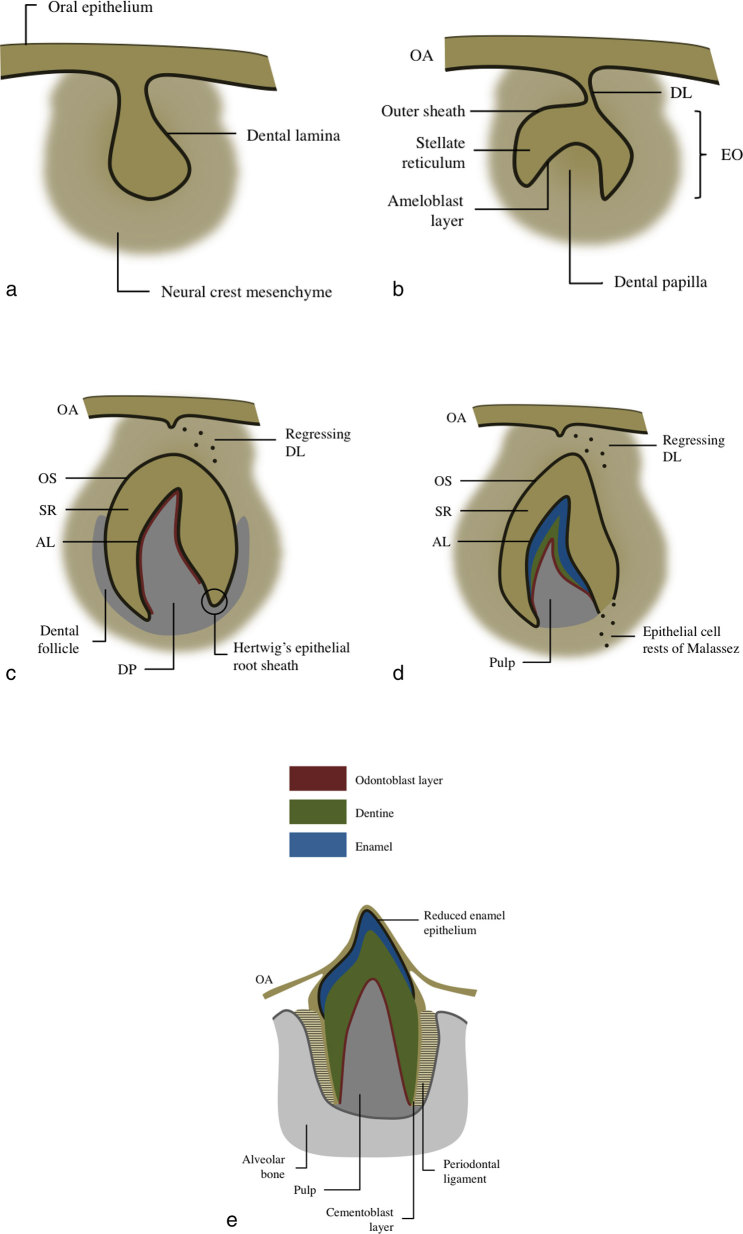
Diagram of tooth development during (**a**) the bud stage (8th week), (**b**) cap stage (10th week), (**c**) bell stage (12th week), (**d**) apposition stage (variable) and (**e**) maturation stage (variable). The primitive oral ectoderm thickens and invaginates to form a C-shaped primary dental lamina. It progressively grows into the subjacent neural crest mesenchyme, giving rise to a bell-shaped tooth primordium. The epithelium of the tooth primordium, known as enamel organ, remains connected to the oral epithelium by a stalk of dental lamina that will soon regress. An outer epithelial sheath, a mesenchyme stellate reticulum and an inner epithelial ameloblast layer constitute the enamel organ. After the ameloblast layer secretes the definitive enamel of the tooth, the reduced enamel epithelium appears superficially, resulting from the fusion of the three primordial layers of the enamel organ. It plays an essential role in the tooth eruption process by secreting connective-tissue-breaking proteases. Deep under the concave surface of the enamel organ, two neural-crest-derived ectomesenchymal structures are separated by the Hertwig’s epithelial root sheath, a double-layered covering of epithelial cells that plays an important role during the tooth root development. While the dental papilla gives rise to the dentin, the dental follicle has the capacity to differentiate into cementoblasts, fibroblasts and osteoblasts, thus giving rise to the cementum. The Hertwig’s epithelial root sheath starts to disintegrate after the deposition of the first dentine layer, leading to the formation of residual epithelial filaments known as epithelial cell rests of Malassez. These are the only odontogenic epithelial cells that may be found in the adult periodontal space.^13–16^ The three recognized sources of epithelial remnants within the mandible are illustrated. AL, ameloblast layer; DL, dental lamina; DP, dental papilla; EO, enamel organ; OA, oral epithelium; OS, outer sheath; SR, stellate reticulum.

Several hypotheses have been postulated to explain the origin of PIOSCC. Epithelial cells have to be present within the bone to give rise to a squamous cell tumour, and the presence of epithelial remnants constitutes the most consensual theory.^8^ The jaws arise from the first pharyngeal arch, which appears on day 22 of embryonic development. It is posteriorly remodelled to form cranial maxillary and caudal mandibular prominences, which give rise to the upper and lower jaws, respectively. Each swelling contains a central cartilage that is produced by neural crest cells. In the mandibular swellings, the central cartilage is known as Meckel’s cartilage. The mandible results from the enlargement and fusion of the bilateral mandibular prominences.^13^ Since Meckel’s cartilage has no epithelium or stem cells that might undergo epithelial differentiation, it cannot be stated as the source of epithelial remnants. Actually, some authors argue in favour of a direct transformation of remnants of the odontogenic epithelium, which may include the remnants of dental lamina in the hard and soft tissue spaces, the epithelial cell rests of Malassez in the periodontal ligament space and the reduced enamel epithelium.^4,5,7,13–16^ Figure 4 summarizes the stages of tooth development and illustrates the three recognized sources of epithelial remnants within the mandible.

The diagnosis of PIOSCC is based on a proper clinical, imaging and histological correlation. None of these parameters may solely provide a secure diagnosis, except when the pathologist is able to recognize the epithelial lining of an odontogenic cyst as the primary site of origin. In all other situations, the diagnosis implies some requisites. First, as PIOSCC arises from the odontogenic epithelium with no initial connection to the oral mucosa, no ulcers should be observed on physical examination. Otherwise, the possibility of an oral mucosal origin cannot be ignored. Second, metastatic SCC ought to be excluded. Since there are no specific histopathological features that can distinguish a metastatic SCC from a PIOSCC, clinical and imaging correlation is mandatory.^4^

Orthopantomography and CT imaging are commonly chosen for the initial imaging approach. Both techniques use ionizing radiation and usually suffice in the diagnosis of tooth-related tumours. The imaging findings of the majority of benign cysts are typical and usually offer no doubt, unless invasive features or multiple lesions are found. In the setting of aggressive lesions, CT is more accurate than orthopantomography by allowing volumetric acquisitions and multiplanar reconstruction. CT is then able to detect small cortical erosions, periosteal reaction and soft tissue calcified components. MRI remains a second-line choice in the evaluation of tooth-related diseases. However, despite being less accurate in the detection of calcified matrices, it better depicts soft tissue involvement and perineural invasion owing to its higher contrast resolution.^13^

The imaging appearance of PIOSCC is variable, ranging from well-defined benign-like masses to ill-defined and often bone-destructing lytic lesions.^9^ Although more typical of tumours such as metastatic SCC or sarcomatous tumours, bone-forming PIOSCCs have also been described. Some authors have reported small radiopaque foci within these tumours that were due to calcification or dentinoid structures. Ground-glass opacity has also been reported, potentially mimicking fibrous dysplasia or ossifying fibroma.^11^ In this case we report a prominent interrupted type of periosteal reaction apart from a medullary new bone formation. This kind of periosteal reaction suggests an aggressive malignancy such as sarcoma, and is not commonly found in metastases or odontogenic tumours. A Codman’s triangle was also found, corresponding to a segment of the periosteum that was elevated from the cortex by the underlying rapidly growing tumour.^17^

As previously stated, PIOSCC is often found in association with odontogenic cysts, so particular attention should be provided to patients with a previous diagnosis of probably benign jaw lesion who present with new symptoms and volumetric changes upon physical examination. Malignant transformation within an odontogenic cyst should be suspected on imaging modalities if the typical unilocular, homogeneous cyst increases in size or develops hazy borders or bone erosions.^8^

This article reports a peculiar imaging presentation of a rare tumour. The imaging findings of an aggressive bone-centred tumour with irregular periosteal reaction are more often seen in a sarcoma or a primary malignant odontogenic tumour. The above described ossification pattern is uncommon in the setting of an SCC, either primary oral or metastatic. With regard to the PIOSCC, calcified components, central ossification and periosteal reaction usually tend to be minimal or absent. The diagnosis of PIOSCC is challenging not only for clinicians and radiologists, but also for pathologists. Even histologically the distinction between a PIOSCC and a metastatic or an oral SCC may be impossible. If a mucosal lesion is identified, the diagnosis will remain uncertain unless the pathologist clearly recognizes an underlying odontogenic cyst. In the absence of both mucosal lesions upon physical examination and imaging evidence of metastatic disease, a histologically proven SCC should be classified as a primary intraosseous tumour.

## Learning points

1. PIOSCC is a rare malignant tumour.
2. PIOSCC may arise from an odontogenic cyst.
3. The exclusion of primary oral mucosa lesions and metastatic disease is mandatory.
4. The imaging appearance of PIOSCC ranges from well-defined masses to ill-defined and bone-destructing lytic lesions.
5. Ossification and irregular periosteal reaction are uncommon in the setting of an SCC.

## Consent

Informed consent was obtained.
